# Medical Association of Georgia

**Published:** 1884-02-20

**Authors:** 


### *MEDICAL ASSOCIATION OF GEORGIA.*

*The Georgia Medical Association*—Will assemble the present year at Macon, Georgia, on the third Wednesday of April next, April 16, 1884.

Attention is invited to the following letter, which has been addressed by Dr. Gray, the Secretary, to the members of the Georgia Medical Association :

ATLANTA, GA., February 12, 1884.

DEAR DOCTOR—Allow me to remind you that the next annual meeting of the Medical Association of Georgia, which convenes at Macon on April 16, is not very far off. I would respectfully request that you favor us with whatever papers, reports of cases, etc., you can, and also see others in your locality and request them to try and be present. I hope we will all thus combine to make this a most interesting and profitable meeting.

